# Exercise‐induced increase in M2 macrophages accelerates wound healing in young mice

**DOI:** 10.14814/phy2.15447

**Published:** 2022-10-05

**Authors:** Makoto Kawanishi, Katsuya Kami, Yukihide Nishimura, Kohei Minami, Emiko Senba, Yasunori Umemoto, Tokio Kinoshita, Fumihiro Tajima

**Affiliations:** ^1^ Department of Rehabilitation Medicine Wakayama Medical University Wakayama Japan; ^2^ Department of Rehabilitation, Wakayama Faculty of Health Care Sciences Takarazuka University of Medical and Health Care Wakayama Japan; ^3^ Department of Rehabilitation Medicine Iwate Medical University Morioka Japan; ^4^ Department of Physical Therapy Osaka Yukioka College of Health Science Ibaraki Japan

**Keywords:** angiogenesis, M2 macrophage, macrophage phenotype, moderate‐intensity exercise, wound healing

## Abstract

Moderate‐intensity exercise performed during wound healing has been reported to decrease inflammatory cytokines and chemokines and accelerate wound healing. However, its effect on macrophage phenotype and the mechanism by which exercise accelerates wound healing remain unclear. The purpose of this study was to investigate the effect of exercise on macrophage phenotype during wound healing and to clarify the relationship between angiogenesis and wound healing. 12‐week‐old male C57BL/6J mice were divided into sedentary (*n* = 6) and exercise groups (*n* = 6). The exercise group performed moderate‐intensity treadmill running exercise (9.0 m/min, 60 min) for 10 days. Double immunofluorescence analysis was performed using F4/80^+^ inducible nitric oxide synthase (iNOS)^+^ for M1 macrophages, F4/80^+^ transforming growth factor‐beta (TGF‐β)1^+^ for M2 macrophages, and CD31^+^ alpha smooth muscle actin (α‐SMA)^+^ for angiogenesis. The exercise group showed significantly accelerated wound healing compared with the sedentary group. From early wound healing onward, exercise significantly inhibited M1 macrophage infiltration and increased M2 macrophage count. Exercise also significantly increased angiogenesis. Furthermore, the M2 macrophage phenotype was significantly correlated with angiogenesis in the exercise group, indicating that M2 macrophages and angiogenesis are related to accelerated wound healing. These findings suggest that moderate‐intensity exercise increases TGF‐β1 derived from M2 macrophages, which may be associated with enhanced angiogenesis and wound healing in young mice.

## Graphical abstract

Moderate‐intensity exercise suppressed M1 macrophage‐derived iNOS and increased M2 macrophage‐derived TGF‐β1 in young mice. In addition, the increase in M2 macrophages may be associated with wound healing and angiogenesis.

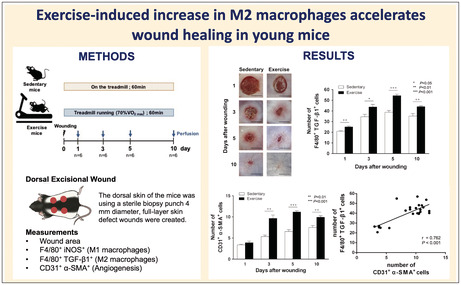

## 1 INTRODUCTION

Delayed wound healing prolongs hospitalization, increases medical costs, and significantly reduces quality of life owing to the pain and distress experienced by the individual (Forbes et al., 2010; Phillips et al., 1994). In addition, decrease in physical activity can lead to decrease in the patient's activities of daily living (Hirase et al., 2018), thereby creating a negative loop that contributes to inactivity and worsening of the condition. Therefore, prevention of delay in wound healing and promotion of healing would benefit patients in many ways. Treatment pertaining to wound healing is thus an important research topic.

Wound healing consists of three processes, depending on the time course: inflammation, proliferation, and tissue remodeling (Baum & Arpey, 2005; Gurtner et al., 2008; Sorg et al., 2017). During the inflammatory phase, neutrophils, macrophages, and lymphocytes infiltrate the wound and produce various cytokines, cell growth factors, and reactive oxygen species (Reinke & Sorg, 2012). During the tissue formation phase, vascular endothelial cells, fibroblasts, and myofibroblasts accumulate and initiate angiogenesis, which is followed by extracellular matrix (ECM) formation (Frykberg & Banks, 2015). During the maturation phase, fibroblasts and myofibroblasts are replaced by collagen fibers, which in turn are replaced by normal tissue, forming the ECM and leading to completion of wound healing (Singer & Clark, 1999).

Macrophages are one of the major cell types associated with wound healing, including the process of angiogenesis (Greaves et al., 2013; Mahdavian Delavary et al., 2011; Rodero & Khosrotehrani, 2010). The macrophages mobilized in wounded tissues are classified into phenotypes such as proinflammatory M1 and anti‐inflammatory M2 macrophages (Murray & Wynn, 2011; Pence & Woods, 2014). M1 macrophages produce and release proinflammatory cytokines (i.e., IL‐1b, IL‐6, and TNF‐α) to induce inflammation. M2 macrophages are involved in migration and proliferation of fibroblasts, keratinocytes, and vascular endothelial cells by producing and releasing anti‐inflammatory cytokines and growth factors (the IL‐1 receptor antagonist [IL‐1Ra], IL‐10, transforming growth factor‐beta [TGF‐β]1, and basic fibroblast growth factor [bFGF]) (Boniakowski et al., 2017; Krzyszczyk et al., 2018; Mosser & Edwards, 2008). Furthermore, TGF‐β1 produced by M2 macrophages plays an important role in regulating differentiation at all stages of wound healing and is an important growth factor that not only alleviates inflammation but also regulates angiogenesis, granulation tissue formation, and ECM remodeling (Hesketh et al., 2017; Jones & Ricardo, 2013).

Only few studies have reported that exercise accelerates wound healing (Emery et al., 2005; Keylock et al., 2008; Zogaib & Monte‐Alto‐Costa, 2011). Emery et al. reported that a group of healthy older adults who participated in a bicycle ergometer exercise program for 3 months at an exercise intensity of 70% of the maximum heart rate expected for their age showed 30% faster wound healing than the sedentary group (Emery et al., 2005). Furthermore, in an animal study, Keylock et al. reported that moderate exercise for 8 days prior to wounding in old BALB/cByJ mice (18‐month‐old) produces significantly greater wound healing compared to the sedentary mice. In addition, they found significant decreased levels of inflammatory cytokines and chemokines in exercised old mice compared with control mice. These results suggested that the improved wound healing response in the old mice may be the results of an exercise‐induced anti‐inflammatory response in the wound (Keylock et al., 2008). Another study demonstrated that moderate‐intensity exercise accelerates wound healing in young C57BL/6 mice (8 week old) and increases α‐smooth muscle actin (α‐SMA) in wounds (Zogaib & Monte‐Alto‐Costa, 2011). These findings indicated that moderate‐intensity exercise accelerates wound healing in both aged as well as young mice. However, the effects of exercise on macrophage phenotype and angiogenesis which may play important roles as possible mechanisms in wound healing processes have been never examined in young mice. Other studies have reported that moderate‐intensity exercise accelerates phenotypic switching from M1 to M2 macrophages in the adipose tissue of obese mice (Kawanishi et al., 2010). Based on these findings, we hypothesized that exercise would induce changes in the macrophage phenotype and accelerates angiogenesis and wound healing during the normal wound healing in young mice. The purpose of this study was to assess the wound healing, macrophage phenotype, and angiogenesis in young mice by subjecting them to moderate‐intensity exercise during the normal wound healing process and to determine the relationship between the effects of exercise and these parameters.

## 2 METHODS

### 2.1 Murine dorsal excisional wound model

Male 12‐week‐old C57BL/6 J mice (SLC) were used in this study. Four mice were housed per cage under a 12‐h light/dark cycle with free access to food and water. Dorsal excisional wound model mice were operated on 1 h after exercise. The mice were deeply anesthetized with 3% anesthetic isoflurane administered continuously by mask at a flow rate of 1–2 L/min to maintain a pain‐free state. The dorsal skin of the mice was shaved with an electric shaver and cleaned with 70% ethanol. Then, using a sterile biopsy punch (BP‐40F; Kai Industries), four symmetrical, 4 mm diameter, full‐layer skin defect wounds were created on the dorsal skin. After surgery, mice were monitored in individual gauges and returned to their usual gauges after awakening. No any post‐wound treatments were applied on mice after surgery. However, several symptoms such as reduction of movement and body weight, swelling, excessive inflammation, and suppuration were never observed in mice. All the experiments were approved by the Animal Care Committee of Wakayama Medical University. All experiments were performed in compliance with the National Institutes of Health Guide for the Care and Use of Laboratory Animals (NIH Publication No. 99‐158, revised 2002).

### 2.2 Experimental groups and treadmill exercise protocol

**FIGURE 1:**
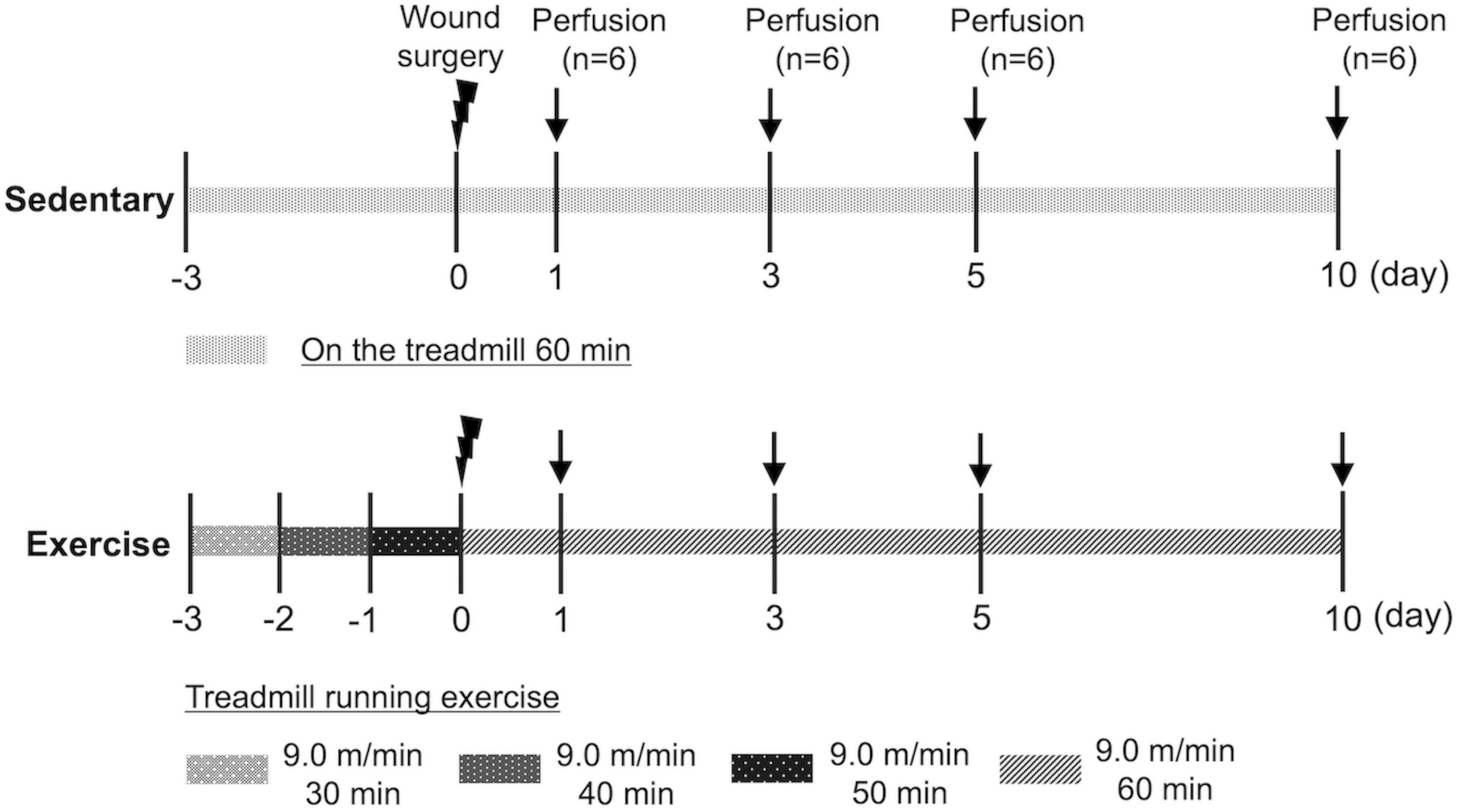
Experimental protocol. The sedentary group was given no food and water during exercise, placed on a treadmill without exercise, and exposed to the same environment as the exercise group for 60 min. The exercise group started treadmill running exercise 3 days before wounding to get acclimated to the exercise. The mice were made to run at a speed of 9.0 m/min for 30 min 3 days before wounding, for 40 min 2 days before wounding, and for 50 min 1 day before wounding. From the day of wounding, the mice were allowed to run for 60 min at a speed of 9.0 m/min for 10 days. In both groups, the mice were sacrificed at 1, 3, 5, and 10 days after wounding (*n* = 6 on days 1, 3, 5, and 10, respectively). Perfusion fixation was performed 1 h after exercise, and skin tissue was harvested.

The exercise protocol is shown in Figure 1. The mice were randomized allocated to two groups: sedentary (*n* = 6) and exercise (*n* = 6) groups. The running exercise was performed on a mouse treadmill (KN‐73‐RM5; Natsume). Treadmill running exercise was initiated for the exercise group 3 days before wounding so that they could get acclimated to the exercise. Exercise was initiated at 6:00 a.m., which was the active phase of the mice. All the mice were allowed to acclimate to the treadmill belt for 10 min before starting the exercise. During the treadmill running exercise, we encouraged the mice to keep running and to maintain their pace along the treadmill belt by gently pushing their tail or hips with our hands. The mice readily responded to this stimulus; to avoid stress‐induced bias, we disconnected the electric shock system, which forced the mice to run. The treadmill inclination was set to 0°. The mice were made to run at a speed of 9.0 m/min for 30 min at 3 days before wounding, for 40 min at 2 days before wounding, and for 50 min at 1 day before wounding. From the day of wounding, the mice were allowed to run for 60 min at a speed of 9.0 m/min for 10 days. This aerobic exercise is considered to be an exercise intensity corresponding to 70% of maximal oxygen uptake (VO_2_ max) for C57BL/6J mice (Ferreira et al., 2007; Høydal et al., 2007; Schefer & Talan, 1996). The sedentary group was deprived of food and water during the exercise sessions and was placed on the treadmill for similar exposure to the noise and vibration for 60 min of the treadmill without exercising. Wound area was evaluated in the sacrificed group (*n* = 6) on day 10 post wounding.

### 2.3 Wound area evaluation

Wound size was recorded daily with a digital camera using an 8 mm spot as the reference. Wound area was analyzed in terms of a percentage of the wound area noted immediately after injury on the basis of photographs obtained using the ImageJ software (version 2.10; National Institutes of Health).

### 2.4 Antibodies

The primary antibodies used in this study were as follows: F4/80 (rat monoclonal antibody; dilution, 1:200; ab6640; Abcam), inducible nitric oxide synthase (iNOS; rabbit polyclonal antibody; dilution, 1:100; ab15323; Abcam), TGF‐β1 (rabbit polyclonal antibody; dilution, 1:100; ab92486; Abcam), CD31 (rat monoclonal antibody; dilution, 1:100; cat. no. DIA‐310; Dianova), and alpha smooth muscle actin (α‐SMA; mouse monoclonal antibody; dilution, 1:500; ab7817; Abcam).

The secondary antibody used to detect F4/80 and CD31 immunoreactivity was Alexa Fluor 488‐conjugated donkey anti‐rat IgG (dilution, 1:500; ab150153; Abcam). The secondary antibody used to detect iNOS and TGF‐β1 immunoreactivity was Alexa Fluor 594‐conjugated donkey antirabbit IgG (dilution, 1:500; ab150064; Abcam). The secondary antibody used to detect α‐SMA immunoreactivity was Alexa Fluor 594‐conjugated donkey antimouse IgG (dilution, 1:500; ab150108; Abcam).

### 2.5 Immunohistochemical analysis

Wounds from the mice of both sedentary and exercise groups (*n* = 6 on days 1, 3, 5, and 10, respectively) were subjected to immunohistochemical analysis. Wound tissue samples were obtained after perfusion fixation 1 h after exercise on days 1, 3, 5, and 10 after wounding. On the day of sacrifice, the mice were deeply anesthetized with 3% anesthetic isoflurane administered continuously through a mask at a flow rate of 1–2 L/min to maintain a pain‐free state. And the mice were transcardially perfused with 50 ml saline, followed by 80 ml ice‐cold 4% paraformaldehyde in 0.1 M PBS. Dorsal skin was obtained using an 8 mm diameter biopsy punch and placed in the same fixative solution for 2 h at 4°C. The skin was transferred to 30% sucrose (w/v) in 0.1 M PBS for 24 h at 4°C, embedded in OCT compound, and cryoprotected using hexane cooled to −60°C with dry ice. Eight‐micrometer‐thick sections were prepared in a cryostat at −20°C and successively mounted on slides coated with 3‐aminopropyl‐ethoxysilane. To block nonspecific staining, the sections were washed with 0.1 M PBS and incubated in 0.1 MPBS containing 5% normal goat serum, 3% bovine blood albumin, and 1% TritonX‐100 for 90 min at room temperature. The sections for double immunofluorescence staining were simultaneously incubated for 48 h at 4°C with two primary antibodies diluted in 0.1 MPBS containing 5% normal goat serum, 3% bovine blood albumin, and 0.3% TritonX‐100. After the sections were incubated with primary antibodies, they were washed with 0.1 M PBS containing 0.1% TritonX‐100 and then incubated overnight at 4°C with secondary antibodies diluted in 5% normal goat serum, 3% bovine blood albumin, and 0.1 MPBS containing 0.1% TritonX‐100.

To visualize the nuclei, the sections were washed with 0.1 M PBS and sealed in Vectashield inclusion medium containing 4′,6‐diamidino‐2‐phenylindole (DAPI; H‐1200; Vector Labs). Fluorescence signals were detected using a fluorescence microscope (BZ‐X800; Keyence). Negative control sections, which were processed without primary antibodies, had no significant positive immunoreactivity and no macrophage autofluorescence.

### 2.6 Quantitative analysis of Immunopositive cells

M1 macrophages were defined as F4/80^+^ iNOS^+^ double‐immunopositive cells, and M2 macrophages were defined as F4/80^+^ TGF‐β1^+^ double‐immunopositive cells. Angiogenesis was defined as the presence of CD31^+^ α‐SMA^+^‐positive cells. For quantification of F4/80^+^ iNOS^+^, F4/80^+^ TGF‐β1^+^, and CD31^+^ α‐SMA^+^ double‐immunopositive cells, sections included center of wound tissue were immunostained, and four fields at wound margins were selected (two fields from the left and right) and counted positive cells located in these fields. For the F4/80^+^ iNOS^+^ and F4/80^+^ TGF‐β1^+^ cells, the number of positive cells per ×400 field was counted. For angiogenesis, the number of CD31^+^ α‐SMA^+^ cells per ×200 field was determined. Angiogenesis was analyzed in cells larger than 25 μm. The results have been expressed in terms of an average value per four fields and have been considered as the number of immunopositive cells per mouse. All experiments were performed by examiner without prior knowledge on the experimental procedures in order to avoid any bias in the results of the quantitative analysis.

### 2.7 Statistical analysis

Quantitative data have been expressed in terms of mean ± standard error of the mean. Statistical analyses were performed using GraphPad Prism version 6.03 (GraphPad Software). For the comparison between sedentary and exercise mice at multiple time points, two‐way repeated measures ANOVA followed by Dunnett's post thoc test was used. To compare the values between two groups, unpaired Student t test was performed. Statistical significance was set at *p* < 0.05. Pearson's product‐rate correlation analysis was used to determine the relationship among the number of M1 and M2 macrophages, angiogenesis, and the rate of wound closure.

## 3 RESULTS

### 3.1 Exercise accelerated wound healing in mice

**FIGURE 2:**
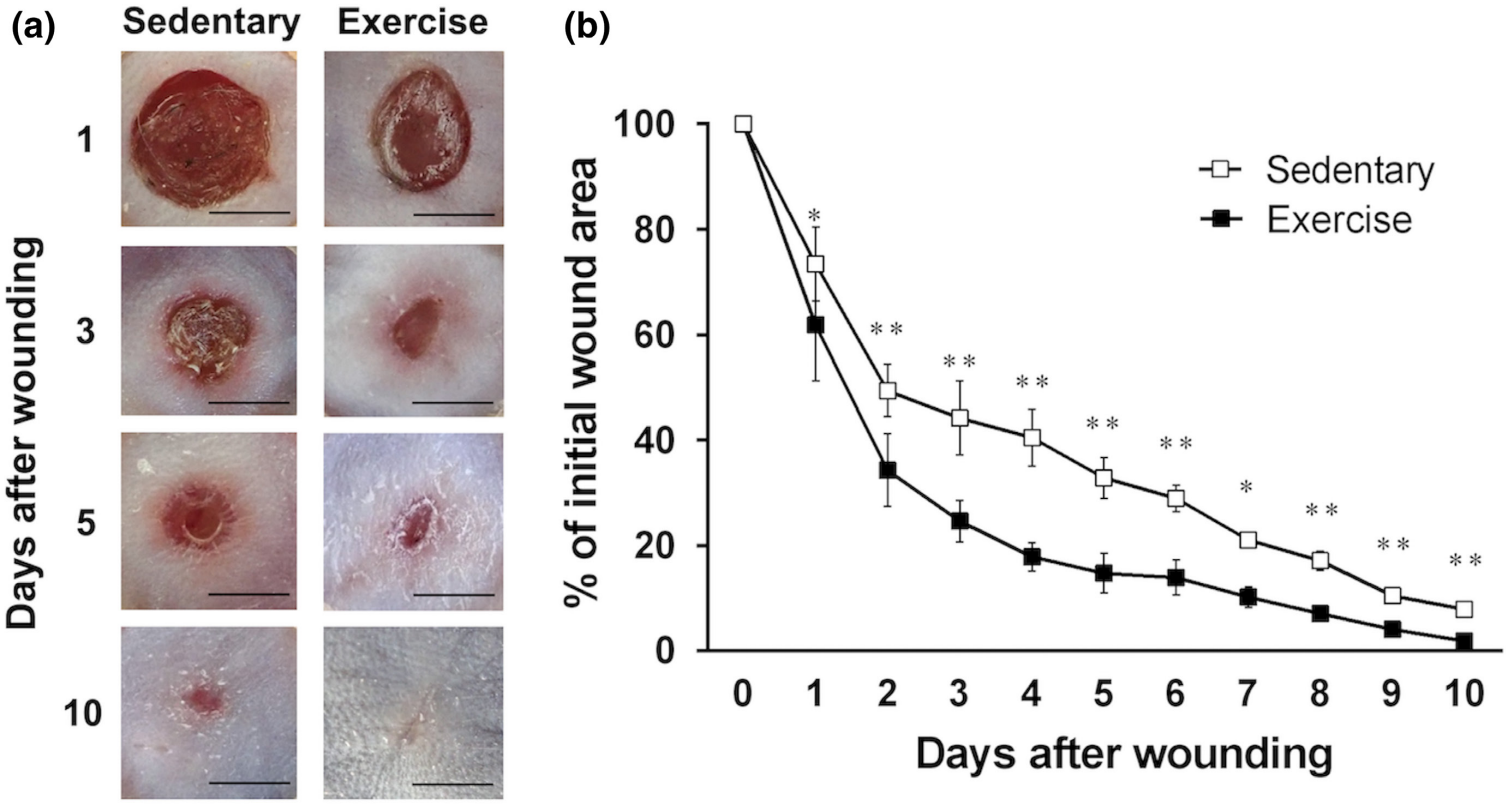
Changes in exercise‐induced wound healing over time. Differences in the wound size of the sedentary and exercise groups. Wound area was evaluated in the sacrificed group (*n* = 6) on day 10 post wounding. (a) Representative results from six individual animals are shown in images. The wounds on the exercising mice were smaller than those on the sedentary mice; this difference was apparent even on observation with the naked eye. Scale bar = 2 mm. (b) Percentage change in wound area at each time point compared with the wound area noted immediately after injury. All values have been expressed in terms of mean ± standard error (*n* = 6). *p < 0.05, **p < 0.01 versus sedentary group

Figure 2a shows wound closure observed with the naked eye. Figure 2b shows wound closure determined in terms of the percentage change in wound area at each time point as compared with the wound area noted immediately after injury. In the exercise group, the wound area was reduced to less than 30% of its original size at 3 days after wounding and the wound almost completely closed at 10 days after wounding (Figure 2b). In the sedentary group, the wound area was 30% of the original size at 6 days after wounding, and the wound was not completely closed at 10 days after wounding (Figure 2b). In particular, wound healing in the exercise group was significantly accelerated compared with that in the sedentary group from 2 to 6 days after wounding (*p* < 0.01).

### 3.2 Treadmill running exercise increased M2 macrophage phenotype

**FIGURE 3:**
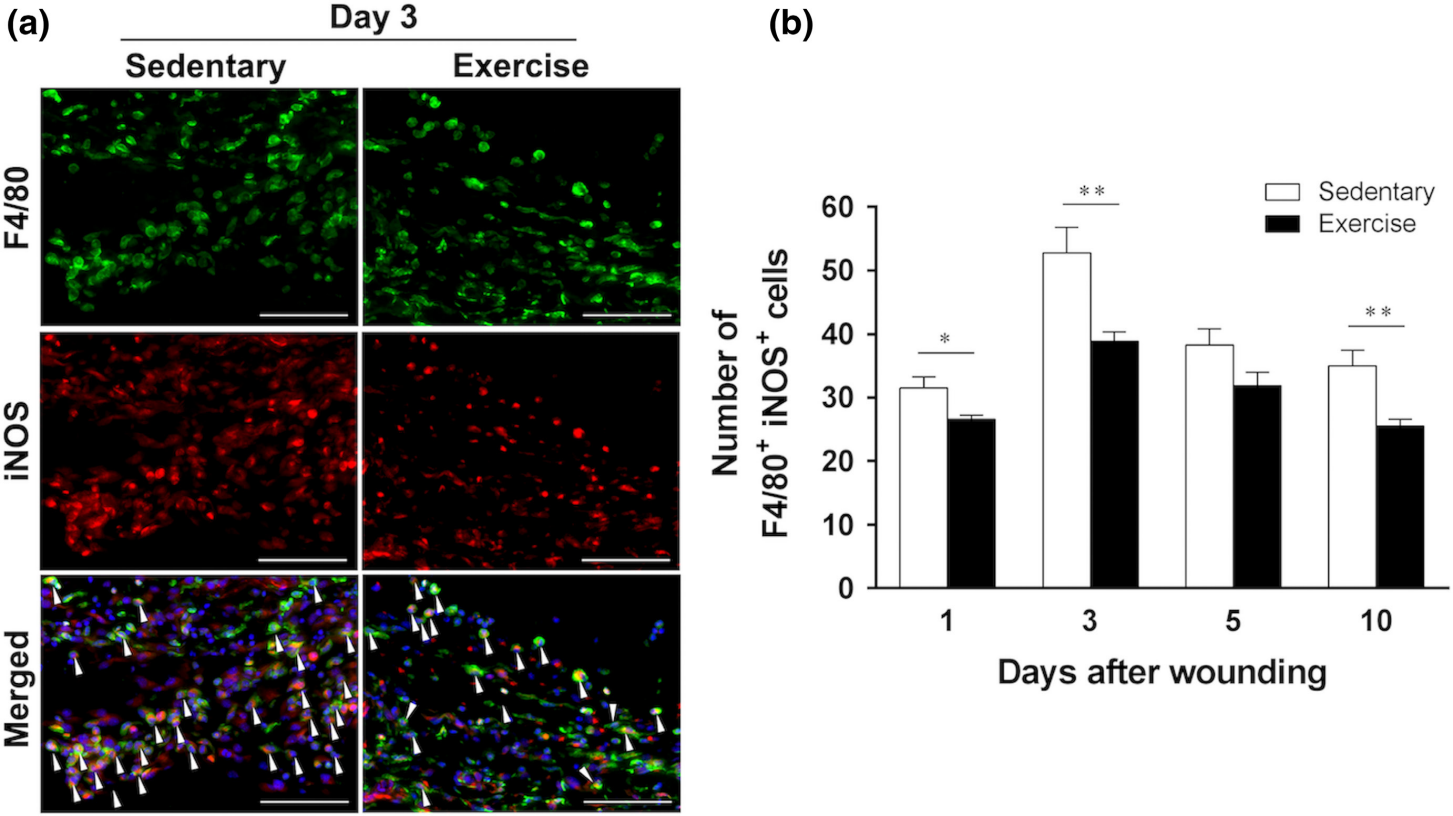
Kinetics of M1 macrophage (F4/80^+^ inducible nitric oxide synthase [iNOS]^+^) expression at skin wound sites. The immunohistochemical results for M1 macrophages at the wound site in the sedentary and exercise groups are shown (*n* = 6 on days 1, 3, 5, and 10, respectively). (a) Representative images from six animals are shown (3 days after wounding). M1 macrophages were identified by double immunofluorescence staining of the F4/80 (green) and iNOS (red) around the nucleus (blue). Scale bar = 100 μm. (b) The number of F4/80^+^ iNOS^+^ cells per field of view in high‐magnification microscopy (original magnification, ×400) is shown. All values have been expressed in terms of mean ± standard error (*n* = 6 per group per time point). Statistical significance was assessed using two‐way ANOVA followed by Bonferroni's post hoc test. The interaction between group and time was significant. Statistical differences between times in the same group are not shown. *p < 0.05, **p < 0.01 versus sedentary group

The images show representative immunofluorescent staining results of six animals at 3 days after wounding (Figure 3a). The number of M1 macrophages peaked 3 days after wounding in both groups and then gradually decreased. At 1 day after wounding, the number of M1 macrophages decreased significantly in the exercise group (26.6 ± 0.6, *p* < 0.05) compared with that in the sedentary group (31.5 ± 1.8). At 3 days after wounding, the number of M1 macrophages significantly decreased in the exercise group (38.9 ± 1.5, *p* < 0.01) compared with that in the sedentary group (52.8 ± 4.0). Furthermore, even at 10 days after wounding, the number of M1 macrophages significantly decreased in the exercise group (25.5 ± 1.0, *p* < 0.01) compared with that in the sedentary group (35.0 ± 2.5) (Figure 3b).

**FIGURE 4:**
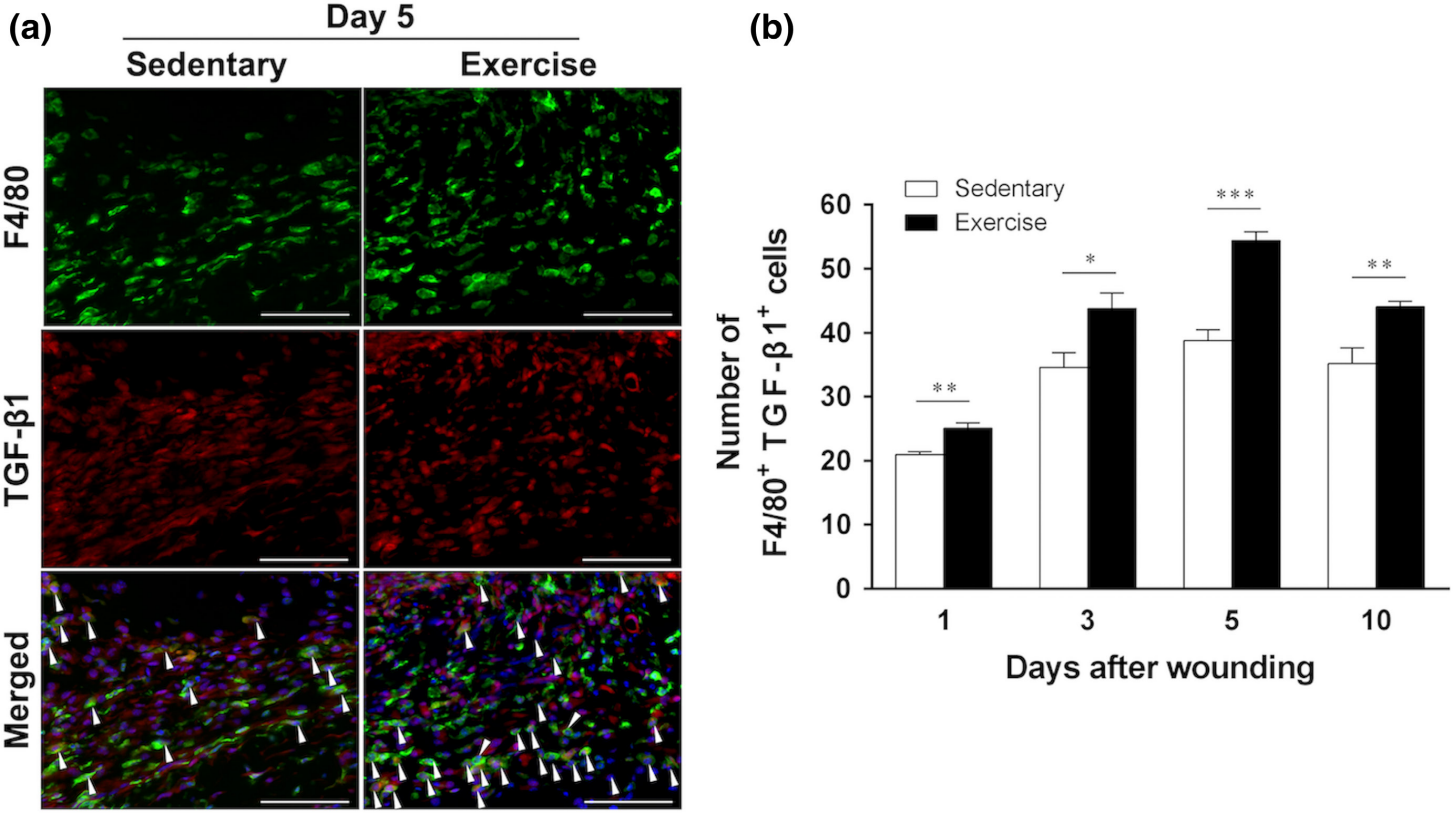
Kinetics of M2 macrophage (F4/80^+^ transforming growth factor‐beta [TGF]‐β1^+^) expression at skin wound sites. The immunohistochemical results for M2 macrophages at the wound site in the sedentary and exercise groups are shown (*n* = 6 on days 1, 3, 5, and 10, respectively). (a) Representative images from six animals are shown (5 days after wounding). M2 macrophages were identified by double immunofluorescence staining of the F4/80 (green) and TGF‐β1 (red) around the nucleus (blue). Scale bar = 100 μm. (b) The number of F4/80^+^ TGF‐β1^+^ cells per field of view in high‐magnification microscopy (original magnification, ×400) is shown. All values have been expressed in terms of mean ± standard error (*n* = 6 per group per time point). Statistical significance was assessed using two‐way ANOVA followed by Bonferroni's post hoc test. The interaction between group and time was significant. Statistical differences between times in the same group are not shown. *p < 0.05, **p < 0.01, ***p < 0.001 versus sedentary group

**FIGURE 5:**
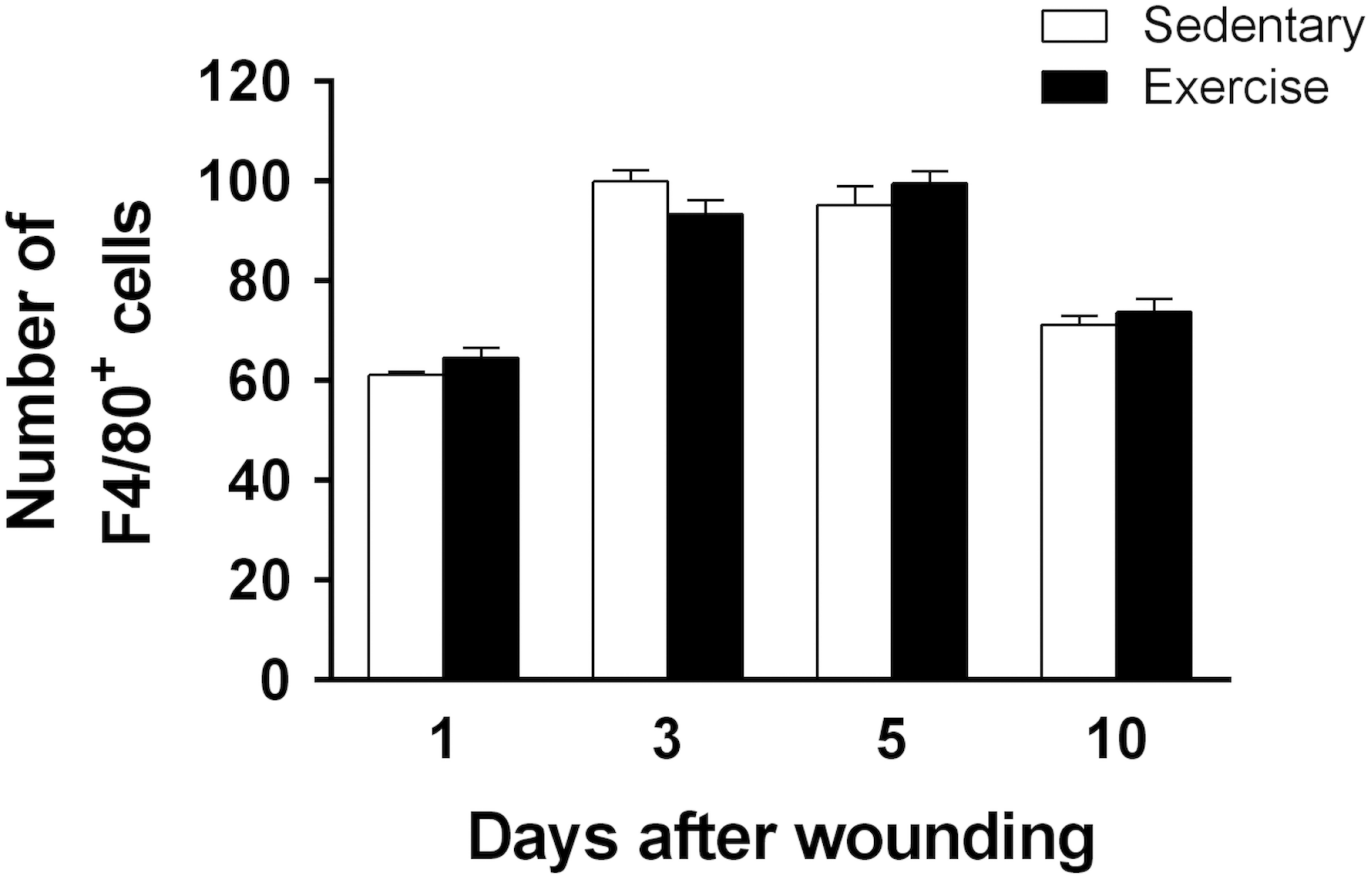
Kinetics of macrophage (F4/80^+^) expression at skin wound sites. The immunohistochemical results for macrophages at the wound site in the sedentary and exercise groups are shown (*n* = 6 on days 1, 3, 5, and 10, respectively). The number of F4/80^+^ cells per field of view in high‐magnification microscopy (original magnification, ×400) is shown. All values have been expressed in terms of mean ± standard error (*n* = 6 per group per time point). Statistical significance was assessed using two‐way ANOVA followed by Bonferroni's post hoc test. The interaction between group and time was not significantly different. Statistical differences between times in the same group are not shown.

The images show representative immunofluorescent staining results of six animals at 5 days after wounding (Figure 4a). The number of M2 macrophages peaked at 5 days after wounding in both groups. At 1 day after wounding, the exercise group (25.0 ± 0.9; *p* < 0.01) had a significantly higher M2 macrophage count than the sedentary group (21.0 ± 0.5). At 3 days after wounding, the number of M2 macrophages in the exercise group (43.8 ± 2.4, *p* < 0.05) increased significantly compared with that in the sedentary group (34.6 ± 2.3). At 5 days after wounding, the number of M2 macrophages peaked in both groups and was significantly higher in the exercise group (25.0 ± 0.9, *p* < 0.001) than in the sedentary group (38.8 ± 1.6). At 10 days after wounding, the number of M2 macrophages decreased in both groups, but increased significantly in the exercise group (44.0 ± 0.9, *p* < 0.01) compared with that in the sedentary group (35.2 ± 2.5) (Figure 4b). Macrophage numbers in both groups showed similar kinetics, increasing until days 3–5 after wounding and then decreasing. At 3 days after wounding, macrophage counts were no significant difference between the exercise group (93.5 ± 6.6) and the sedentary group (99.9 ± 5.4). At 5 days after wounding, there were no significant difference between the exercise group (101.3 ± 5.7) and the sedentary group (95.2 ± 8.7). There was no significant difference in macrophage numbers between the two groups at 1, 3, 5, and 10 days after wounding (Figure 5). Thus, treadmill running exercise suppressed iNOS‐producing M1 macrophage count and increased that of TGF‐β1‐producing M2 macrophages. In addition, exercise significantly increased M2 macrophage count from an early stage, with the count peaking at 5 days after wounding.

### 3.3 Treadmill running exercise increased angiogenesis and accelerated wound healing

**FIGURE 6:**
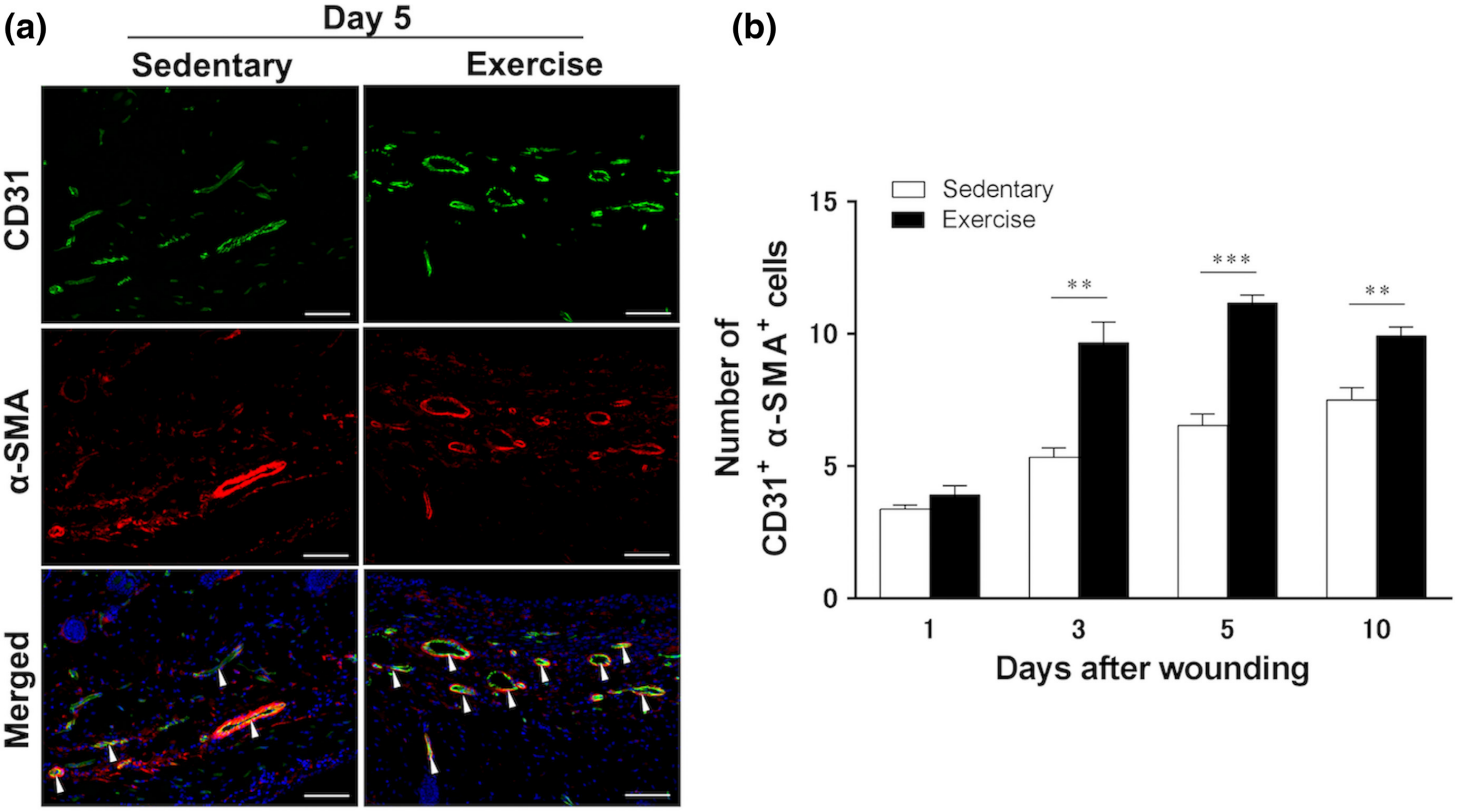
Kinetics of angiogenesis (CD31^+^ alpha smooth muscle Actin [α‐SMA]^+^) expression at skin wound sites. The immunohistochemical results for angiogenesis at the wound site in the sedentary and exercise groups are shown (*n* = 6 on days 1, 3, 5, and 10, respectively). (a) Representative images from six animals are shown (5 days after wounding). Angiogenesis was identified by double immunofluorescence staining of CD31 (green) and α‐SMA (red). Scale bar = 100 μm. (b) The number of CD31^+^ α‐SMA^+^ cells per field of view in high‐magnification microscopy (original magnification, ×200) is shown. All values have been expressed in terms of mean ± standard error (*n* = 6 per group per time point). Statistical significance was assessed using two‐way ANOVA followed by Bonferroni's post hoc test. The interaction between group and time was significant. Statistical differences between times in the same group are not shown. **p < 0.01, ***p < 0.001 versus sedentary group

The images show representative immunofluorescent staining results of six animals at 5 days after wounding (Figure 6a). The exercise group showed early increase in angiogenesis compared with that in the sedentary group and showed peak angiogenesis at 5 days after wounding. At 3 days after wounding, the exercise group showed significantly greater angiogenesis (8.8 ± 0.6; *p* < 0.01) than the sedentary group (5.5 ± 0.3). At 5 days, angiogenesis peaked in the exercise group (10.8 ± 0.3; *p* < 0.001) and increased in the sedentary group (6.9 ± 0.4). At 10 days after wounding, angiogenesis peaked in the sedentary group (7.5 ± 0.5) but was higher in the exercise group (9.9 ± 0.3, *p* < 0.01; Figure 6b).

### 3.4 Exercise‐induced increase in M2 macrophages were associated with accelerated wound healing

**FIGURE 7:**
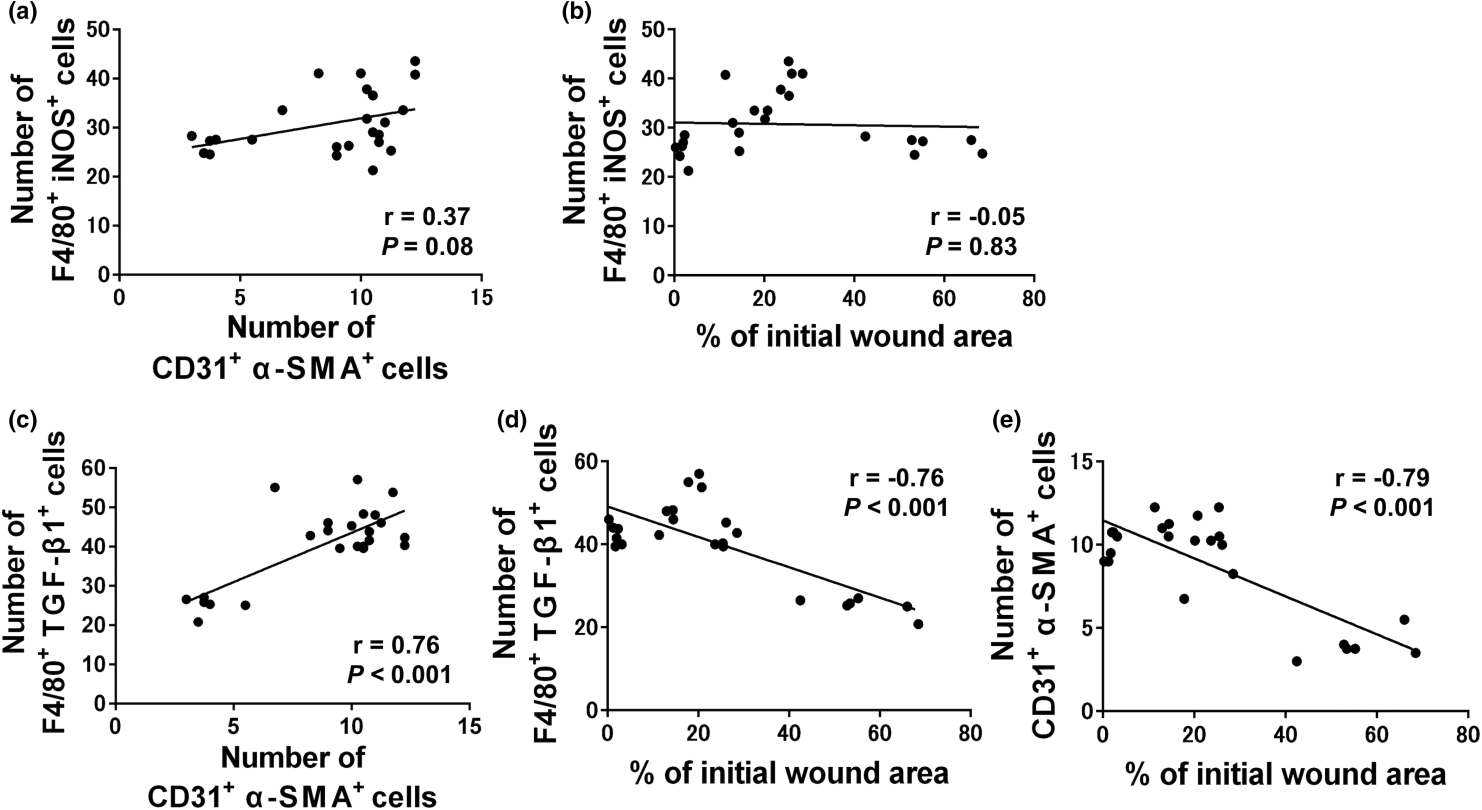
Correlation of macrophages with angiogenesis and wound healing in the exercise group. In the exercise group, we examined the correlation between the number of M1 and M2 phenotype macrophages, wound healing rate, and angiogenesis at 1, 3, 5, and 10 days after wounding. M1 macrophages were defined as F4/80^+^ iNOS^+^ double‐immunopositive cells and M2 macrophages as F4/80^+^ TGF‐β1^+^ double‐immunopositive cells. Angiogenesis was defined as the presence of CD31^+^ α‐SMA^+^‐positive cells. (a) The M1 macrophage count was not significantly correlated with angiogenesis (*n* = 24; *r* = 0.37; *p* = 0.08). (b) The M1 macrophage count was not significantly correlated with the wound closure rate (*n* = 24; *r* = −0.05; *p* = 0.83). (c) The M2 macrophage count showed statistically significant positive correlation with angiogenesis (*n* = 24; *r* = 0.762; *p* < 0.001). (d) The M2 macrophage count showed statistically significant negative correlation with the wound closure rate (*n* = 24; *r* = −0.76; *p* < 0.001). (e) Angiogenesis showed statistically significant negative correlation with wound closure rate (*n* = 24; *r* = −0.79; *p* < 0.001). α‐SMA, alpha smooth muscle actin; iNOS, inducible nitric oxide synthase; TGF‐β1, transforming growth factor‐beta 1

In the exercise group, we examined the correlation between the number of M1 and M2 phenotype macrophages, wound healing rate, and angiogenesis at 1, 3, 5, and 10 days after wounding. The M1 macrophage phenotype was not correlated to angiogenesis (*n* = 24; *r* = 0.37; *p* = 0.08; Figure 7a) or wound healing rates (*n* = 24; *r* = −0.05; *p* = 0.83; Figure 7b). The M2 macrophage phenotype showed significant positive correlation with angiogenesis (*n* = 24; *r* = 0.762; *p* < 0.001; Figure 7c) and significant negative correlation with wound healing (*n* = 24; *r* = −0.76; *p* < 0.001; Figure 7d). Angiogenesis showed significant negative correlation with wound healing (*n* = 24; *r* = −0.79; *p* < 0.001; Figure 7e).

## 4 DISCUSSION

In this study, moderate‐intensity exercise accelerated wound healing in young mice. We also found that it increased the count of the M2 phenotype macrophages and angiogenesis from an early stage of wound healing. Furthermore, we found that M2 macrophages and angiogenesis promoted exercise‐induced wound healing. To our knowledge, our study is the first to examine the relationship between exercise‐induced phenotypic changes in macrophage and angiogenesis during the process of wound healing.

A few studies have previously examined the relationship between exercise and wound healing; of these, some showed that exercise promotes wound healing. Keylock et al. reported that moderate treadmill running exercise (VO_2_ max, 70%, 30 min/day) for 8 days in young mice (3‐month‐old) trends to improve wound healing, although the difference was not significant compared to the sedentary group (Keylock et al., 2008). When C57BL/6 mice (8‐week‐old) were subjected to treadmill running exercise at moderate (VO_2_ max, 70%), high (VO_2_ max, 80%), and strenuous (VO_2_ max, 90%) intensity exercise for 45 min/day for 2 weeks after wounding, the moderate exercise group showed significantly faster wound contraction (7 days after wounding), re‐epithelialization (14 days after wounding), and more rapid wound healing compared with the sedentary mice (Zogaib & Monte‐Alto‐Costa, 2011). These results suggest that the duration and frequency of exercise may affect to wound healing. Therefore, taken together these findings suggest that the time, intensity, and frequency of exercise used in this study are effective in accelerating wound healing in young mice.

There are no reports of exercise and macrophage phenotype changes in wound healing, but there are several reports in mouse adipose tissue. In a previous study, high‐fat diet‐induced obese mice that performed treadmill running exercise (12–20 m/min; 60 min; 5 times a week) for 16 weeks showed lower M1 macrophage‐associated CD11c mRNA and increased M2 macrophage‐associated CD163 mRNA levels than sedentary mice did. TNF‐α mRNA expression decreased in the mice subjected to exercise. Furthermore, they reported that exercise may have inhibited M1 macrophage infiltration into adipose tissue via TLR4 downregulation and induced a phenotypic switch from M1 to M2 macrophages because Toll‐like receptor 4 mRNA, which activates inflammatory responses, had decreased (Kawanishi et al., 2010). Baek et al. studied how lifelong voluntary exercise on a running wheel affected macrophage phenotype in the adipose tissue of C57BL/6 mice. They divided the animals into an exercise group with a running wheel in the rearing gauge and a control group without a running wheel and observed them from 6 to 99 weeks of age. They found that CD206‐positive macrophage (M2 macrophage) count was significantly increased in the exercise group compared with that of CD11c‐positive macrophages (M1 macrophages). Furthermore, the levels of ICAM‐1 and TNF‐α produced by M1 macrophages decreased, that of arginase 1 produced by M2 macrophages increased, and the M1:M2 polarization ratio changed (Baek et al., 2020). The authors speculate that the mechanism underlying the macrophage phenotype change is upregulation of the transcription factor peroxisome proliferator activated receptor (PPAR)‐γ and its activator protein peroxisome proliferator activated receptor‐γ coactivator (PGC)‐1α. During wound healing, PPAR‐γ resolves inflammation by binding to specific DNA. It also inhibits pro‐inflammatory gene expression by binding to other transcriptional modulators (Chawla, 2010; Straus & Glass, 2007). Mirza et al. reported that the switch in macrophage phenotype during wound healing is associated with the upregulation of PPAR‐γ and its downstream target, PGC‐1. Furthermore, in studies using PPAR‐γ knockout mice, loss of PPAR‐γ activity in macrophages was found to prolong inflammation, significantly reduce angiogenesis and granulation tissue, and delay wound healing (Mirza et al., 2015). A previous study reported that mice that performed treadmill running exercise (15 m/min; 30 min/day) 5 days a week for 4 weeks showed significantly higher PPAR‐γ levels in the gastrocnemius muscle than the no‐exercise group (Sasaki et al., 2014). It has been reported that contracted skeletal muscle during exercise works as an endocrine organ and produces myokines. IL‐6, one of the myokines, is released from skeletal muscle into the blood by acute exercise and causes anti‐inflammatory effects by inhibiting TNF‐α and IL‐1β from macrophages and increasing IL‐1ra and IL‐10 (Pedersen et al., 2004). IL‐10 has been reported to promote polarization from M1 to M2 by activating STAT3 via IL‐10 receptor (Porta et al., 2015; Wang et al., 2014). Our findings of phenotypic changes in macrophages may be related to these mechanisms. However, the current study did not examine such signaling receptors or transcription factors, and therefore, the detailed mechanism of macrophage phenotypic changes in wound healing could not be determined; it is possible though that exercise acts as a switch for changes in macrophage phenotype from M1 to M2 during wound healing.

TGF‐β1, which is produced by M2 macrophages, is an important growth factor that regulates the proliferation and differentiation of various types of cells at all wound healing stages (Faler et al., 2006; Klass et al., 2009). It is also reported to be important for calming inflammation and angiogenesis (Okuda et al., 1998; Schmid et al., 1993) and is essential for re‐epithelialization via regulation of granulation tissue formation and ECM remodeling (Mantovani et al., 2013; Shull et al., 1992). TGF‐β1‐knockout mice have been reported to show delayed inflammatory cell infiltration, reduced angiogenesis, defective granulation tissue formation, unorganized apoptosis in wounds, and significant delay in wound healing (Kulkarni et al., 1993; Shull et al., 1992). In addition, macrophage depletion during wound healing was found to lead to decreased TGF‐β1 expression in wounds in mice (Mirza et al., 2009). Jetten et al. reported that increase in M2 macrophages leads to increase in the number of CD31‐positive cells and promotes angiogenesis (Jetten et al., 2014). In our study, CD31^+^ α‐SMA^+^‐positive cells were used as an indicator of angiogenesis. These cells are coated with pericytes and vascular endothelial cells, indicating more mature microvessels (von Tell et al., 2006). We found that exercise promoted more mature angiogenesis and that the M2 macrophage phenotype was correlated to angiogenesis. These findings suggest that the change in macrophage phenotype to M2 after exercise strongly influences promotion of angiogenesis.

In the current study, the relationship among macrophage phenotype, wound healing, and angiogenesis was also examined in the exercise group. Mahdavian et al. reported that macrophages significantly increase within 3–5 days after wounding during the wound healing process (Mahdavian Delavary et al., 2011), which supported the macrophage dynamics in the present study. Keylock et al. found that inflammatory chemokine and cytokine levels were significantly reduced early after wounding (within 5 days) in an exercise group compared with those in controls, but no difference in F4/80 gene expression was found (Keylock et al., 2008). In our study, inflammation‐induced M1 macrophage count was significantly reduced and that of anti‐inflammatory M2 macrophages was significantly increased in the exercise group from early post‐wounding. We speculate that exercise may alter the macrophage phenotype from inflammatory to anti‐inflammatory during early wound healing. In the exercise group, there was no correlation between M1 macrophages and angiogenesis or wound healing, but there was significant correlations between M2 macrophages and angiogenesis and wound healing and between angiogenesis and wound healing. These findings suggest that the change in polarity of M2 macrophages is a more important factor in the mechanism underlying exercise‐induced promotion of wound healing than the decrease in M1 macrophage count.

Studies evaluating the effects of exercise on wound healing have reported a variety of exercise intensity protocols. While some experiments were conducted at low intensities and others at high intensity, Zogaib et al. reported that moderate‐intensity training promoted wound healing in young mice compared to high (VO_2_ max, 80%) and strenuous (VO_2_ max, 90%) intensity (Zogaib & Monte‐Alto‐Costa, 2011). Reports on exercise and immune function in humans have long shown that high‐intensity exercise decreases immune function (Elphick et al., 2003; Gleeson, 2006; Suzuki et al., 1996). In addition, in reports investigating exercise and anti‐inflammatory effects, exercise intensity, exercise duration, and mobilized muscle mass have been found to correlate with immune function and anti‐inflammatory substance secretion (Gleeson, 2007; Gleeson et al., 2011; Petersen & Pedersen, 2005). Thus, the intensity of exercise may affect the degree of wound healing. Therefore, it is important to determine the exercise intensity and duration that will effectively promote the polarity change to M2 macrophages in wound healing in the future.

The current study has some limitations. Although the present study used young normal mice as experimental animals, the effects of exercise on wound healing may be different in older mice or disease model mice. This is a limitation of this study. However, the effects of exercise on changes of macrophage phenotypes during the wound healing process have not been previously examined in young adult male mice. Therefore, the present data may be very valuable to know changes of macrophage phenotypes during normal wound healing processes in growth period. Macrophage phenotypes were classified into two main types: M1 or M2. However, macrophage phenotypes are complex, and it is difficult to divide them into two simple categories. Macrophages are further classified into various subtypes according to their functions such as tissue repair, inflammation calming, and immunomodulation (Mantovani et al., 2013; Murray & Wynn, 2011). Therefore, the current results may not completely reflect the macrophage subset. M1 macrophages express high iNOS levels and induce inflammation. M2 macrophage subtypes express TGF‐β1 and are involved in tissue repair (Barrientos et al., 2008). Further studies are thus required to define a more detailed subset of M2 macrophages. Also, we assessed wound healing by only external measurements, but not histology of epithelialization and granulation tissue formation. This is a limitation of this study. In addition, since skin anatomy, wound healing process, and immune systems differ between rodents and humans, all findings which were obtained from rodents cannot apply to humans. In general, mice skin heals by contraction, whereas human skin heals by re‐epithelialization with keratinocytes covering the granulation tissue of the wound. Chen et al. reported that the wound healing process in mice heals by both skin contraction and re‐epithelialization, which suggested to be a good model of wound healing process (Chen et al., 2015). Therefore, clinical studies should be performed to further understand differences in wound healing across organisms and to determine whether these findings are applied to humans.

In conclusion, the present study indicated the moderate‐intensity exercise suppresses M1 macrophage‐derived iNOS and increases M2 macrophage‐derived TGF‐β1 in young mice. In addition, the increase in M2 macrophages may be associated with wound healing and angiogenesis. There was no change in the number of macrophages in both groups, suggesting that exercise may shift phenotype of macrophages to promote wound healing. Our study may provide clues for future studies targeting motility and macrophage phenotypic changes during wound healing.

## AUTHOR CONTRIBUTIONS

Makoto Kawanishi and Emiko Senba designed the experiments; Makoto Kawanishi, Kohei Minami, and Katsuya Kami performed experiments; Makoto Kawanishi and Yasunori Umemoto contributed to the analysis and interpretation of data; Makoto Kawanishi, Tokio Kinoshita, and Yukihide Nishimura wrote the manuscript; Katsuya Kami and Fumihiro Tajima read and revised the manuscript; and Makoto Kawanishi and Tokio Kinoshita performed all figure drawings.

## CONFLICT OF INTEREST

The authors declare no conflict of interest.

## ETHICS STATEMENT

All experiments were performed in accordance with the National Institutes of Health Guide for the Care and Use of Laboratory Animals and received ethical approval from the Wakayama Medical University Animal Protection Committee.
